# Prevalence of Plasmid‐Mediated Quinolone Resistance Genes in *Escherichia coli* Isolates From Colonic Biopsies of Iranian Patients With Inflammatory Bowel Diseases: A Cross‐Sectional Study

**DOI:** 10.1002/hsr2.70204

**Published:** 2024-12-17

**Authors:** Samira Alipour, Mina Owrang, Mohsen Rajabnia, Meysam Olfatifar, Hossein Kazemian, Hamidreza Houri

**Affiliations:** ^1^ Foodborne and Waterborne Diseases Research Center, Research Institute for Gastroenterology and Liver Diseases Shahid Beheshti University of Medical Sciences Tehran Iran; ^2^ Faculty of Medical Science, Sari Branch Islamic Azad University Sari Iran; ^3^ Non‐Communicable Diseases Research Center Alborz University of Medical Sciences Karaj Iran; ^4^ Gastroenterology and Hepatology Diseases Research Center Qom University of Medical Sciences Qom Iran; ^5^ Clinical Microbiology Research Center Ilam University of Medical Sciences Ilam Iran

**Keywords:** ciprofloxacin, *Escherichia coli*, inflammatory bowel disease, plasmid‐mediated quinolone resistance

## Abstract

**Background and Aims:**

Emerging evidence suggests that ciprofloxacin and other quinolones can be effectively used as adjuncts to immunosuppressive therapy in managing inflammatory bowel disease (IBD). Clinical isolates of Enterobacterales frequently exhibit quinolone resistance. Additionally, increased IBD severity has been linked to the proliferation of Enterobacterales in the gut. This study aimed to explore the frequency of fluoroquinolone resistance and the presence of associated resistance genes in *Escherichia coli* isolates obtained from intestinal biopsies of patients with IBD in Iran.

**Methods:**

In this research, we conducted a study that involved the isolation and examination of *E. coli* bacteria from inflamed ileal and/or colonic tissues of patients diagnosed with IBD, specifically ulcerative colitis (UC) and Crohn's disease (CD), during colonoscopy procedures. We collected demographic and clinical information from the patients. To identify *E. coli* strains that were resistant to quinolone antibiotics, we performed both phenotypic and molecular analyses.

**Results:**

From the colonic and ileal biopsies of 121 patients with IBD, we isolated 107 unique strains of *E. coli*. Among these strains, 18 (16.8%) were derived from patients with CD, and 89 (83.2%) came from those with UC. Antimicrobial susceptibility tests revealed that 61 out of 107 isolates (57%) of the isolates showed phenotypic resistance to at least one type of quinolone. Additionally, plasmid‐mediated quinolone resistance (PMQR) genes, specifically *oqxA*, *oqxB*, and *qnrS* were detected in the *E. coli* strains linked to both UC and CD. Notably, there was a significant positive correlation observed between intestinal colonization by ciprofloxacin‐resistant *E. coli* and the patients' history of extended ciprofloxacin antibiotic therapy.

**Conclusion:**

Our results reveal that a significant number of patients with IBD carry quinolone‐resistant *E. coli*. This colonization may pose a risk factor that could affect disease progression and contribute to potential complications.

**Abbreviations**
AIEC: adherent‐invasive *E. coli*
CD: Crohn's disease
CDAI: Crohn's disease activity index
ESBL: extended‐spectrum β‐lactamase
IBD: inflammatory bowel diseases
MDR: multidrug resistant
PMQR: plasmid‐mediated quinolone resistance
UC: ulcerative colitis

## 1 Introduction

Inflammatory bowel diseases (IBDs), including ulcerative colitis (UC) and Crohn's disease (CD), are chronic, progressive, and relapsing immune‐mediated gut disorders of unknown etiology [1]. The exact cause of IBD remains unknown, but several key factors are implicated in its pathogenesis. These include genetic variations, dysregulation of the immune response, alterations in the intestinal microbiota, and environmental influences [2, 3]. The influence of host genetics on disease risk appears to account for only a small portion of the overall variability, highlighting the significance of environmental factors, including the gut microbiota [4]. Several authors have reported that the development of IBD may relate to an immunological reaction to gut microbiota or an unidentified bacterial pathogen in genetically susceptible individuals [5]. There is considerable evidence suggesting that CD may arise from the invasion of the intestinal mucosa by certain bacterial strains known as pathobionts. This invasion is characterized by an inadequate response from the innate immune system, leading to the proliferation of bacteria within local macrophages [6]. In contrast, for UC, there is limited evidence to support the idea of bacteria invading as intact organisms. Instead, current research favors the hypothesis that a compromised mucosal barrier could facilitate greater interactions between bacterial components and basolateral receptors, resulting in inflammation [7].

The prevailing notion is that dysbiosis, characterized by the disrupted balance of gut microbial communities, plays a significant role in the development and advancement of IBD. Multiple studies indicate that heightened disease activity in both CD and UC is linked to the proliferation of Enterobacterales in the gastrointestinal (GI) tract [8]. Furthermore, the disturbance of the intestinal microbiota due to antibiotic use has long been recognized as a potential factor triggering the onset of IBD [9]. Certain *Escherichia coli* types with specific virulence properties, known as adherent‐invasive *E. coli* (AIEC), have been frequently identified in patients with CD ileitis [10]. Furthermore, for more than two decades, the overrepresentation of specific *E. Coli* strains has been described in UC patients [11, 12].

In the realm of IBD therapeutics, the conventional approach involves the administration of nonspecific immune‐suppressive measures, comprising a diverse range of anti‐inflammatory, immunosuppressant agents, and antibiotics [13]. Antibiotic agents are thought to function through the eradication or reduction of bacterial immune targets, potentially exerting favorable immunomodulatory effects [14]. Given the notable adverse effects associated with prolonged use of immunosuppressive drugs and steroids, antibiotics are often recommended as a supplement to immunosuppressant therapy to enhance treatment outcomes, combat secondary GI infections, and facilitate remission induction in IBD patients. Over the past decades, numerous studies have investigated the effect of several antimicrobial agents alone or in combination, as a single treatment or in adjunct to standard therapy in both CD and UC patients, however, the results have been conflicting [15, 16]. The diversity in trial designs, types of antibiotics employed, endpoints assessed, and concurrent medications utilized has rendered the comparison of studies challenging and the application of results into clinical practice cumbersome. Consequently, the utilization of antibiotics in IBD management is currently delimited to specific contexts outlined in international guidelines.

In the context of flare‐ups in severe cases of UC and colonic and perianal CD, the addition of a combination of oral ciprofloxacin and metronidazole has demonstrated its efficacy as a beneficial complement to immunosuppressive therapy [14, 17]. Existing evidence suggests that ciprofloxacin and quinolones possess immunomodulating properties in addition to their antimicrobial effects [18]. Furthermore, previous studies have underscored the important role of ciprofloxacin in managing secondary complications of both CD and ulcerative colitis UC, including abscesses and gastrointestinal infections [19]. Empirical evidence further supports the effectiveness of ciprofloxacin in treating active IBD [20]. However, it is crucial to note that local epidemiological data indicate a high prevalence of ciprofloxacin‐ and quinolone‐resistant Enterobacterales colonization in IBD patients [21]. Our own research revealed a higher occurrence of ciprofloxacin‐resistant *E. coli* among individuals who had previously received ciprofloxacin therapy for IBD flare‐ups compared to those without such treatment [22]. This suggests that prolonged exposure to ciprofloxacin may contribute to the proliferation of ciprofloxacin‐resistant Enterobacterales, potentially impacting the progression of IBD. The transfer of horizontally transferable elements carrying genes encoding quinolone resistance could facilitate the overrepresentation of potentially harmful resistant bacteria like *E. coli* and *Klebsiella pneumoniae* in the presence of quinolones, leading to the selection and dissemination of quinolone‐resistant strains [23]. Additionally, plasmid‐mediated horizontally transferable resistance genes may explain the connection between quinolone resistance and resistance to other agents [24]. While there are limited reports on plasmid‐mediated transferable quinolone resistance among *E. coli* isolates from IBD patients, our study aims to investigate fluoroquinolone resistance patterns and associated plasmid‐mediated transferable genetic markers in *E. coli* strains isolated from colonic biopsies of Iranian individuals diagnosed with IBD.

## 2 Methods and Materials

### 2.1 Patients and Sample Collection

Our research adhered to the STROBE (Strengthening the Reporting of Observational Studies in Epidemiology) guidelines for reporting observational studies [25]. Approval for the study was obtained from the Ethical Review Committee of Ilam University of Medical Sciences in Ilam, Iran (Approval No. IR.MEDILAM.REC.1401.154). Respect for ethical and regulatory standards was maintained throughout the study. Participation in the study was voluntary, and no compensation or incentives were provided to the participants. Before the colonoscopy procedure, written informed consent was obtained from all individuals involved, ensuring their understanding and agreement to participate. This cross‐sectional study included 121 patients with IBD treated at Taleghani Hospital, Tehran, between July 2022 and August 2023. The diagnosis and classification of IBD were based on endoscopic, histopathologic, and clinical criteria. Biopsy samples were collected from inflamed regions of the ileum and/or colon during the colonoscopy. Demographic and clinical data for each participant were recorded using a structured questionnaire.

### 2.2 Bacterial Isolation and Identification

Colonic biopsy specimens were obtained using sterile forceps during colonoscopy and immediately placed in brain heart infusion (BHI) broth (Merck, Darmstadt, Germany). These samples were then transported to the microbiology laboratory at the Research Institute for Gastroenterology and Liver Diseases. Upon receipt, the biopsies were homogenized and cultured on MacConkey agar (Merck, Darmstadt, Germany), and incubated overnight at 37°C under aerobic conditions. Lactose‐fermenting colonies were selected for further identification. *E. coli* isolates were identified using conventional biochemical tests, followed by molecular confirmation through PCR amplification of the *phoA* gene with specific primers as described previously [26].

### 2.3 Antimicrobial Susceptibility Testing

We performed in vitro susceptibility testing of fluoroquinolone antibiotics using the Kirby‐Bauer disk diffusion method, following the guidelines outlined by the Clinical and Laboratory Standards Institute (CLSI). The antibiotic disks utilized in the testing comprised ciprofloxacin (5 µg), ofloxacin (5 µg), levofloxacin (5 µg), moxifloxacin (5 µg), and gatifloxacin (5 µg).

### 2.4 Detection of Plasmid‐Mediated Quinolone Resistance (PMQR) Genes

Genomic DNA from *E. coli* isolates was extracted using the QIAamp DNA Stool Mini Kit (Qiagen, Hilden, Germany) following the manufacturer's protocol. The concentration and purity of the extracted DNA were measured with a NanoDrop ND‐1000 spectrophotometer (Thermo Scientific, Waltham, MA, USA). Isolated DNA was stored at −20°C until used for molecular assays.

The detection of PMQR genes (*qepA*, *qnrs*, *qnrD*, *qnrA*, *qnrC*, *qnrB*, *aac(6′)‐Ib*, *oqxB*, and *oqxA*) was performed using specific primers (see Table 1). Each PCR reaction consisted of 12.5 µL of Taq DNA Polymerase Master Mix (Ampliqon, Denmark), 10 picomoles/µL of each primer, and 1 µL (~100 ng) of DNA template, with a total volume of 25 µL. The PCR conditions were: initial denaturation at 96°C for 5 min, followed by 35 cycles of denaturation at 96°C for 30 s, annealing at primer‐specific temperatures (Table 1), extension at 72°C for 45 s, and a final extension at 72°C for 10 min. PCR products were visualized using electrophoresis on a 1.5% agarose gel stained with ethidium bromide.

**Table 1:** Primers used for genotypic detection of quinolone resistance.

Primer	Sequence (5′–3′)	Target	*T*_m_ (°C)	Product size (bp)	Reference
qnrA‐F	AGAGGATTTCTCACGCCAGG	*qnrA*	57	619	[27]
qnrA‐R	GCAGCACTATKACTCCCAAGG				
qnrB‐F	GGMATHGAAATTCGCCACTG	*qnrB*	57	264	[28]
qnrB‐R	TTTGCYGYYCGCCAGTCGAA				
qnrC‐F	GGGTTGTACATTTATTGAATC	*qnrC*	57	447	[29]
qnrC‐R	TCCACTTTACGAGGTTCT				
qnrD‐F	CGAGATCAATTTACGGGGAATA	*qnrD*	57	282	[30]
qnrD‐R	AACAAGCTGAAGCGCCTG				
qnrS‐F	GCAAGTTCATTGAACAGGCT	*qnrS*	57	428	[28]
qnrS‐R	TCTAAACCGTCGAGTTCGGCG				
qepA‐F	CTGCAGGTACTGCGTCATG	*qepA*	60	403	[31]
qepA‐R	CGTGTTGCTGGAGTTCTTC				
oqxA‐F	GACAGCGTCGCACAGAATG	*oqxA*	62	339	[27]
oqxA‐R	GGAGACGAGGTTGGTATGGA				
oqxB‐F	CGAAGAAAGACCTCCCTACCC	*oqxB*	62	240	[27]
oqxB‐R	CGCCGCCAATGAGATACA				
aac‐F	TTGCGATGCTCTATGAGTGGCTA	*aac(6′)‐Ib*	57	482	[32]
aac‐R	CTCGAATGCCTGGCGTGTTT				

### 2.5 Statistical Analysis

We utilized R software (version 4.0.3, The R Foundation for Statistical Computing, Vienna, Austria) to perform data analysis. Spearman's correlation test was utilized to assess the associations between variables, considering *p* < 0.05 as statistically significant. Moreover, to obtain a more comprehensive understanding of the factors contributing to resistance and account for confounding variables, we conducted multivariate logistic regression. To enhance the clarity and prevent misinterpretation of our data, we followed the guidelines outlined by Assel et al. [33]. Initially, we conducted descriptive analysis, presenting categorical variables as percentages and continuous variables as means along with their standard deviations. To gain deeper insights into the effects of factors such as prior CIP therapy (yes/no), disease type (UC/CD), disease activity, NSAID use, immunosuppressant use, and anti‐TNF drug use on resistance (yes/no), we performed the following analytical strategies. Spearman's correlation test was employed to assess the relationships between variables, employing a two‐sided hypothesis and considering *p* < 0.05 as statistically significant. The correlation coefficient ranges from −1 to 1, where values closer to −1 or 1 indicate stronger correlations, while values closer to zero suggest negligible correlation. Furthermore, we employed univariate and multivariate logistic regression to elucidate these relationships. Each variable was first analyzed separately in the logistic model to determine its unadjusted effect, followed by a combined analysis to report the adjusted effects.

We conducted data analysis using R software, utilizing packages such as “rcompanion,” “ggplot2,” and “ggcorrplot.”

## 3 Results

### 3.1 Characteristics of the Patients and *E. coli* Isolates

In this study, we enrolled 121 patients, including 99 with UC and 22 with CD. The median ages were 36.08 ± 14.37 years for UC patients and 39.32 ± 17.22 years for CD patients. Among the UC patients, 27 (27.3%) had mildly active disease (total Mayo score ≤ 5), 38 (38.4%) had moderately active disease (total Mayo Score 6–9), and 34 (34.3%) had severely active disease (total Mayo score > 9). In the CD group, 8 (36.4%) had mildly active disease (total CDAI ≤ 220) and 14 (63.6%) had moderately active disease (total CDAI 220–400). Table 2 provides a detailed summary of the demographic and clinical characteristics of IBD patients. From the colonic and ileal biopsies of these 121 patients, we isolated 107 individual *E. coli* strains: 89 from UC patients and 18 from CD patients.

**Table 2:** Baseline demographic and characteristics of patients with IBD enrolled in this study.

Variable	Ulcerative colitis (*n* = 99)	Crohn's disease (*n* = 22)
**Age, median (years) (±IQR)**	36.08 ± 14.37	39.32 ± 17.22
**Male, *n* (%)**	46 (46.4%)	12 (54.5%)
**Female, *n* (%)**	53 (53.5%)	10 (45.4%)
**Ethnicity**		
**Persians**	43 (43.4%)	12 (54.6%)
**Turks**	24 (24.2%)	5 (22.7%)
**Kurds**	11 (11.1%)	4 (18.2%)
**Arab‐Persians**	4 (4.1%)	0 (0%)
**Lurs**	9 (9.1%)	1 (4.5%)
**Gilaks**	8 (8.1%)	0 (0%)
**Smoking, *n* (%)**	16 (16.1%)	6 (27.2%)
**Disease activity**		
**Flare‐up**	89 (89.8%)	14 (63.6%)
**Remission**	10 (10.1%)	8 (36.3%)
**IBD medications**		
**Mezalasine**	41 (41.4%)	5 (22.7%)
**Sulfasalazine**	18 (18.1%)	4 (18.1%)
**Azathioprine**	22 (22.2%)	4 (18.1%)
**CinnoRA**	7 (7.07%)	1 (4.5%)
**Prednisolone**	11 (11.1%)	9 (9.09%)
**Infliximab**	7 (7.07%)	0 (0%)
**Bloody stool**	76 (76.7%)	12 (54.5%)
**Prior CIP Therapy**		
**Ciprofloxacin**	14 (14.1%)	3 (13.6%)
**Metronidazole**	24 (24.2%)	2 (9.09%)
**Metronidazole, Ciprofloxacin**	14 (14.1%)	9 (40.9%)

### 3.2 Quinolone Resistance Among IBD‐Associated *E. coli*

**Figure 1:**
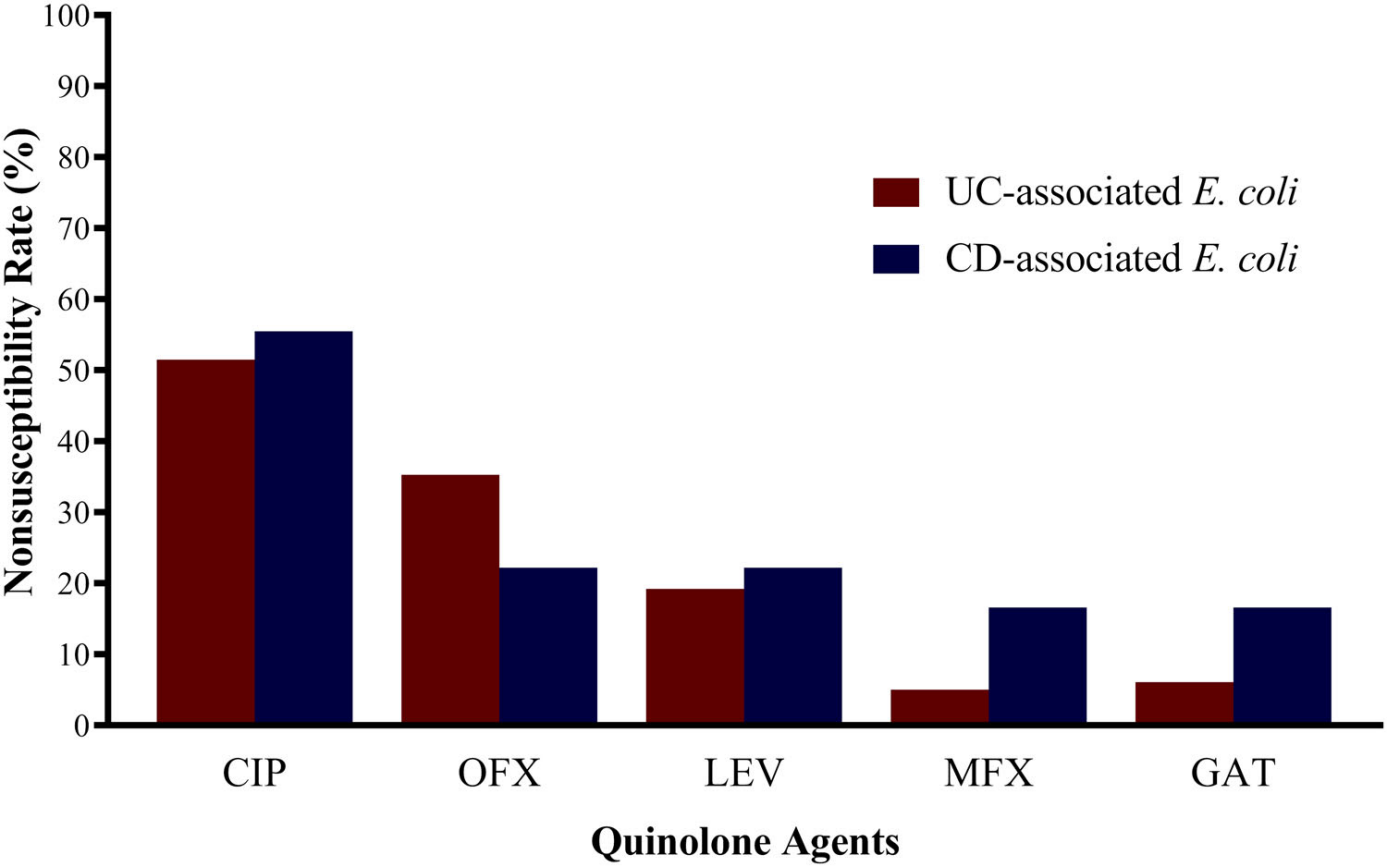
Quinolone resistance of colonic *E. coli* isolates from colonic biopsies of IBD patients. CD, Crohn's disease; CIP, ciprofloxacin; GAT, gatifloxacin; LEV, levofloxacin; MFC, moxifloxacin; OFX, ofloxacin; UC, ulcerative colitis.

Antimicrobial susceptibility testing showed that 61 out of 107 isolates (57%) were phenotypically non‐susceptible to ciprofloxacin, with 47 (43.9%) fully resistant and 14 (13%) intermediate‐resistant. Figure 1 illustrates the quinolone non‐susceptibility results of *E. coli* isolates from UC and CD patients. PCR testing of quinolone‐resistant isolates detected the presence of quinolone resistance genes *qnrS*, *oqxA*, and *oqxB* in both UC and CD‐associated *E. coli*. As detailed in Table 3, UC‐associated *E. coli* carried *qnrS* (*n* = 9), *oqxA* (*n* = 14), *oqxB* (*n* = 10), *oqxA* + *oqxB* (*n* = 5), and *qnrS* + *oqxA* (*n* = 3), whereas CD‐associated *E. coli* carried *qnrS* (*n* = 1), *oqxA* (*n* = 2), *oqxB* (*n* = 1), and *qnrS* + *oqxA* (*n* = 1). Table 3 also presents the prevalence of PMQR genes among IBD‐related *E. coli* isolates.

**Table 3:** Occurrence and frequency of PMQR determinants among IBD‐related *E. coli* isolates according to the resistance to quinolone agents.

PMQR gene Quinolone Resistance	UC‐associated *E. coli* (*n* = 99)					CD‐associated *E. coli* (*n* = 22)			
	*qnrS* (*n* = 9)	*oqxA* (*n *= 14)	*oqxB* (*n *= 10)	*oqxA+oqxB* (*n *= 5)	*qnrS+ oqxA* (*n* = 3)	*qnrS* (*n* = 1)	*oqxA* (*n* = 2)	*oqxB* (*n *= 1)	*qnrS+ oqxA* (*n *= 1)
**CIP**	9	14	10	5	3	1	2	2	2
**OFX**	6	8	7	4	2	1	2	2	2
**LEV**	3	1	2	2	3	1	1	1	1
**MFX**	1	1	1	1	1	1	1	1	1
**GAT**	1	2	1	1	1	1	1	1	1

**Figure 2:**
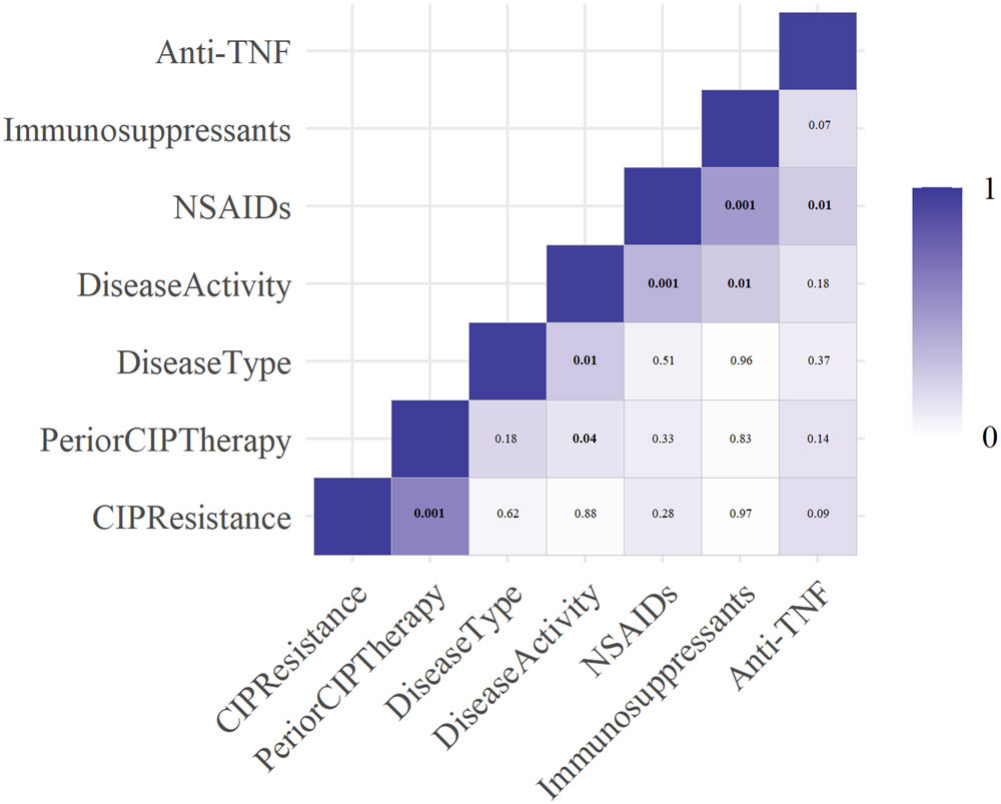
Heatmap illustrating the pairwise correlation between ciprofloxacin resistance and demographic and clinical characteristics of patients with IBD calculated with Pearson's correlation. The deeper the color represents the higher the positive correlation and the numbers indicate the *p* values.

Spearman's correlation analysis identified a significant positive correlation (*r* = 0.8; *p* < 0.001) between the presence of ciprofloxacin‐resistant *E. coli* in the intestines and prior therapy with ciprofloxacin (Figure 2). This finding suggests that the inappropriate use of quinolones may facilitate the proliferation of resistant strains in the guts of patients with IBD. However, subsequent multivariate logistic regression analysis showed no significant correlation between ciprofloxacin‐resistant *E. coli* and prior ciprofloxacin therapy (odds ratio: 1.08; 95% confidence interval: 0.46−2.52; *p* < 0.001). Additionally, no significant relationships were observed with other demographic and clinical variables (Table 4).

**Table 4:** Logistic regression analysis for resistance to quinolones according to other demographical and clinical data.

Variables	Unadjusted OR (95% CI)	*p* value	Adjusted OR (95% CI)	*p* value
**Prior CIP therapy**	1.08 (0.46−2.52)	0.85	0.94 (0.43−2.07)	0.89
**Disease type**	1.9 (0.69−5.19)	0.2	1.96 (0.77−5.01)	0.15
**Disease activity**	1.21 (0.37−3.96)	0.33	1.46 (0.53−4.04)	0.45
**Using NSAIDs**	1.02 (0.39−2.65)	0.95	1.12 (0.53−2.37)	0.76
**Using immunosuppressants**	0.96 (0.36−2.52)	0.93	1.03 (0.45−2.33)	0.93
**Using anti‐TNF drugs**	1.02 (0.3−3.47)	0.78	1.18 (0.37−3.71)	0.77

Abbreviations: CI = confidence interval; OR = odds ratio.

## 4 Discussion

According to the American College of Gastroenterology (ACOG) guidelines in 2009, although antibiotics are frequently employed for the treatment of UC, their efficacy for the disease course has not been consistently demonstrated by research studies [34]. However, in cases of GI infections or abscesses, appropriate antibiotic therapy or drainage is necessary [34]. In 2011, the British Society of Gastroenterology (BSG) guidelines released there is some evidence supporting that quinolones could be beneficial in treating secondary complications of CD such as abscesses and bacterial overgrowth [35]. In addition, perianal complications of CD and simple fistulas should be addressed with ciprofloxacin (500 mg orally twice daily for 4 to 8 weeks) or levofloxacin (500–750 mg once daily for 4–8 weeks) either alone or in combination with metronidazole (10–20 mg/kg/day orally for 4–8 weeks) [36]. Moreover, it has been found that the utilization of a combination of anti‐TNF therapy and ciprofloxacin could provide greater benefits than using each agent separately. Studies have shown that using a combination of infliximab and ciprofloxacin or adalimumab and ciprofloxacin is more effective than using either anti‐TNF agent alone for treating fistulas and reducing surgical fistula drainage [37, 38]. However, frequent use of quinolones has recently been cautioned due to the possibility of experiencing tendonitis, tendon rupture, neuropathy, and importantly, the potential emergence and spread of antibiotic resistance both within the gut of the individual and the broader population.

We specifically focused on PMQR genes due to their crucial role in the horizontal transfer of resistance genes among different bacterial species. We concentrated on plasmid‐mediated mechanisms because they can rapidly spread within the gut microbiota and hospital environments. Furthermore, plasmid‐mediated resistance elements are a key factor in the strong associations observed between quinolone resistance and resistance to other antibiotics. This aspect is particularly important for understanding and managing the spread of antibiotic resistance in patients with IBD, who frequently experience recurrent hospitalizations. As far as we know, only a minuscule portion of research has been conducted to determine quinolone resistance in Enterobacteriaceae strains that colonized the intestinal mucosa of IBD patients. In a study conducted by Dogan et al., it was found that *E. coli* isolates from CD patients frequently manifest resistance to ciprofloxacin compared to those isolated from the intestine of non‐IBD individuals [39]. In a recent study in Iran, about half of IBD patients harbored *E. coli* isolates resistant to ciprofloxacin and ofloxacin [40], which is in accordance with our findings that 57% of isolates exhibited non‐susceptibility to ciprofloxacin. More importantly, we found a positive correlation between the harboring of ciprofloxacin‐resistant *E. coli* and prior long‐term quinolone therapy among IBD patients. A commonly held belief is that having received quinolone previously is a risk factor for the selection of resistant strains and the development of clinically significant resistance, and using these agents repeatedly can raise the probability of therapeutic failure [41, 42]. These findings indicate that prophylactic strategies involving ciprofloxacin may pose significant challenges and might not be a viable long‐term option for most patients with IBD. As an alternative, combination antimicrobial therapy could be considered for IBD patients who do not have contraindications to its use. For example, Breton et al. reported that a regimen combining ciprofloxacin with two or three other oral antibiotics achieved a clinical response in 63.5% of patients and clinical remission in 39.7%, with a relatively low incidence of side effects [43]. Beyond the improvements in clinical response and remission rates, we observed independent enhancements in disease biomarkers. Similarly, Flanagan et al. reported that hydroxychloroquine exhibited synergistic effects when used with doxycycline and ciprofloxacin, both of which are effective against intracellular AIEC, a suspected pathogen in the development and progression of IBD [44]. In cases of quinolone resistance, other broad‐spectrum antibiotics may be recommended for managing IBD complications. For example, Nishikawa et al. found that a combination of amoxicillin, fosfomycin, and metronidazole therapy not only induced remission but also proved suitable for long‐term maintenance of UC, with fewer and milder side effects [45].

PMQR determinants are already broadly distributed geographically, usually carried on ESBL‐producing bacteria. The horizontal intergenus exchange of plasmids carrying PMQRs and ESBLs simultaneously, as well as the antibiotic selection of these genes due to the long exposure, could lead to an overrepresentation of resistant bacterial populations in the gut, and hence, result in conferring resistance to almost all clinically important antimicrobial agents. Unfortunately, we found that 41.4% of UC‐ and 27.7% of CD‐associated *E. coli* isolates harbored at least one PMQR determinant, mainly *oqx* genes. The *oqx* and *qnr* genes have been found frequently in clinical isolates of the Enterobacterales from Iran [46, 47], a region where high levels of resistance to ciprofloxacin and other quinolones are reported regularly. To the best of our knowledge, there is insufficient data regarding the prevalence of IBD patients carrying intestinal PMQR‐producing bacteria. Our findings revealed that the *oqxA* gene was the most prevalent PMQR identified in patients with both UC and CD. We did not detect *qnrA*, *qnrB*, *qnrC*, *qnrD*, *qepA*, and *aac(6')‐Ib*.

In this study, some limitations need to be acknowledged. One limitation pertained to the lack of investigation into mutation‐based resistance, such as the *gyrA* mutation. By restricting our analysis solely to PCR detection, we overlooked other mechanisms contributing to resistance, such as point mutations which could be identified through comprehensive whole‐genome sequencing analyses. Additionally, as a cross‐sectional study, we were unable to ascertain whether quinolone resistance originated from antibiotic pressure selection or the community. Therefore, our future objective is to conduct a follow‐up study to assess quinolone resistance at the time of IBD diagnosis in new cases, as well as to conduct screening throughout the disease progression. By undertaking such cohort studies, we aim to address the timing of quinolone resistance acquisition more definitively.

Our study findings reveal a concerning association between the presence of quinolone‐resistant *E. coli* strains and a substantial portion of patients diagnosed with both UC and CD. These results raise alarms about a potential risk factor linked to the progression and complications of these diseases. We also detected a substantially high prevalence of PMQR determinants among *E. coli* isolates from Iranian IBD patients. As described previously, most of the PMQR‐producing Enterobacteriales also carried other resistance genes leading to a multidrug resistance phenotype, and hence, these findings stress the expansion of the gut resistome in patients with IBD. The risk from the overrepresentation of PMQR‐producing *E. coli* needs to be taken seriously, especially when ciprofloxacin has a crucial role in treating the course of IBD and its complications. It is important to highlight that a majority of quinolone‐resistant *E. coli* was isolated from IBD patients who had previously received ciprofloxacin for the long term. Therefore, the use of ciprofloxacin in managing IBD may be a double‐edged sword, and it is necessary to conduct further extensive studies to investigate the impact of antibacterial therapy with ciprofloxacin, individually or in combination with other medications. Moreover, determining the antibiotic resistance profile of fecal *E. coli* helps recognize appropriate antimicrobial therapies in IBD patients. Furthermore, the field of personalized medicine that leverages the resistome for managing antibiotic‐resistant GI infections is a burgeoning area of investigation. This approach centers on customizing antibiotic treatments to suit individual patients by examining their distinct gut microbiota and the corresponding antibiotic‐resistance genes. By concentrating on specific microbiota characteristics and resistance profiles, healthcare providers can formulate more effective and precision‐driven therapeutic strategies. However, advancing this methodology necessitates additional research and the acquisition of comprehensive, individualized data to better assess and standardize dietary interventions and their functional roles in a more tailored manner.

## Author Contributions

**Samira Alipour:** investigation, validation, methodology, writing–original draft, visualization. **Mina Owrang:** methodology, investigation, resources, writing–review and editing. **Mohsen Rajabnia:** writing–original draft, visualization, project administration, resources, data curation, investigation. **Meysam Olfatifar:** formal analysis, data curation. **Hossein Kazemian:** funding acquisition, conceptualization, resources, writing–review and editing, validation, methodology, project administration. **Hamidreza Houri:** conceptualization, methodology, data curation, supervision, resources, writing–review and editing, project administration, validation.

## Ethics Statement

This study was reviewed and approved by the Ethical Review Committee of the Ilam University of Medical Sciences, Ilam, Iran (No. IR.MEDILAM.REC.1401.154).

## Consent

The patients/participants provided their written informed consent to participate in this study.

## Conflicts of Interest

The authors declared no conflicts of interest.

## Transparency Statement

The lead author Hossein Kazemian and Hamidreza Houri affirms that this manuscript is an honest, accurate, and transparent account of the study being reported; that no important aspects of the study have been omitted; and that any discrepancies from the study as planned (and, if relevant, registered) have been explained.

## Data Availability

Data sharing is not applicable to this article as no data sets were generated or analyzed during the current study. All data generated or analyzed during this study are included in this published article.
